# Highly multiplexed tissue imaging using repeated oligonucleotide exchange reaction

**DOI:** 10.1002/eji.202048891

**Published:** 2021-03-10

**Authors:** Julia Kennedy‐Darling, Salil S. Bhate, John W. Hickey, Sarah Black, Graham L. Barlow, Gustavo Vazquez, Vishal G. Venkataraaman, Nikolay Samusik, Yury Goltsev, Christian M. Schürch, Garry P. Nolan

**Affiliations:** ^1^ Department of Microbiology & Immunology Stanford University School of Medicine Stanford CA USA; ^2^ Department of Pathology Stanford University School of Medicine Stanford CA USA; ^3^ Department of Bioengineering Stanford University Stanford CA USA; ^4^ Department of Pathology and Neuropathology University Hospital and Comprehensive Cancer Center Tübingen Tübingen Germany; ^5^ Akoya Biosciences 1505 O'Brien Drive Menlo Park CA USA; ^6^ Becton Dickinson San Jose CA USA

**Keywords:** CODEX, DNA‐conjugated antibodies, Multiplexed tissue imaging, Single‐cell analysis, Spatial single‐cell biology

## Abstract

Multiparameter tissue imaging enables analysis of cell‐cell interactions in situ, the cellular basis for tissue structure, and novel cell types that are spatially restricted, giving clues to biological mechanisms behind tissue homeostasis and disease. Here, we streamlined and simplified the multiplexed imaging method CO‐Detection by indEXing (CODEX) by validating 58 unique oligonucleotide barcodes that can be conjugated to antibodies. We showed that barcoded antibodies retained their specificity for staining cognate targets in human tissue. Antibodies were visualized one at a time by adding a fluorescently labeled oligonucleotide complementary to oligonucleotide barcode, imaging, stripping, and repeating this cycle. With this we developed a panel of 46 antibodies that was used to stain five human lymphoid tissues: three tonsils, a spleen, and a LN. To analyze the data produced, an image processing and analysis pipeline was developed that enabled single‐cell analysis on the data, including unsupervised clustering, that revealed 31 cell types across all tissues. We compared cell‐type compositions within and directly surrounding follicles from the different lymphoid organs and evaluated cell‐cell density correlations. This sequential oligonucleotide exchange technique enables a facile imaging of tissues that leverages pre‐existing imaging infrastructure to decrease the barriers to broad use of multiplexed imaging.

## Graphical abstract

We simplified the multiplexed imaging method CO‐Detection by indEXing (CODEX) by employing cyclic stripping and annealing of fluorescent oligonucleotides to DNA‐barcoded antibodies. We stained five human lymphoid tissues with a panel of 46 antibodies. This enabled quantitative spatial single‐cell biology analysis of differential cell‐type composition of immune follicles.

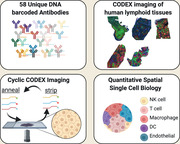

## Introduction

Identifying cells and cell phenotype through antibody binding to known proteins is a fundamental tool in basic research and clinical practice. Spatial information provided by cellular localization is critical for understanding tissue homeostasis and characterizing disease [1, 2, 3, 4, 5, 6, 7, 8, 9, 10, 11]. Currently, immunohistochemistry and immunofluorescence imaging are the workhorses for defining cellular and subcellular protein organization for pathologists and biologists. These modalities allow a limited number of proteins to be investigated simultaneously; however, simultaneous characterization of cell phenotype with multiple proteins for many cell types has been shown necessary to predict therapeutic outcomes [8, 9, 10]. Additionally, the characterization and identification of tissue substructures (e.g. tertiary lymphoid structures) by simultaneous identification of multiple cell types or discrete cell subsets is important, since such have been associated with disease susceptibility and immunotherapy response [1, 2, 3, 4, 5, 6, 7].

Recently, several imaging technologies have been developed that have multiplexing capabilities of flow or mass cytometry (beyond 30 markers). These techniques enable detailed cataloguing of location and coordination of cellular phenotypes, cell‐cell interactions, and larger cellular ensembles. Methods based on mass cytometry employ isotope‐labeled antibodies and use raster laser ablation (imaging mass cytometry [IMC]) [12] or ion beams (multiplexed ion beam imaging [MIBI]) for imaging [13, 14]. Other techniques employ matrix‐assisted laser desorption/ionization (MALDI) trapped ion‐mobility spectrometry (TIMS) [15] or Raman scattering or vibrational signatures of chemical bonds to perform multiplexed imaging [16], yet these techniques lack resolution, are limited in multiplexing abilities, or require specialized instruments and expertise.

Most advanced optical multiplexing methods are limited by spectral overlap restricting simultaneous detection to ten markers or fewer [17]. To overcome these limitations, techniques like multiepitope‐ligand cartography (MELC) [18], multiplexed fluorescence microscopy (MxIF) [19], tissue‐based cyclic immunofluorescence (t‐CyCIF) [20], and iterative indirect immunofluorescence imaging (4i) [21] use cyclic immunofluorescence imaging protocols involving fluorophore inactivation or antibody stripping and restaining. Because each cyclic stripping or bleaching step requires a substantial time (hours), increasing the number of parameters linearly increases the amount of time necessary for one tissue.

Another approach uses DNA‐barcoded antibodies that are visualized by cyclic addition and removal of fluorescently labeled DNA probes. Techniques based on this principle include exchange‐points accumulation in nanoscale topography (PAINT) [22], DNA exchange imaging (DEI) [23], immunostaining with signal amplification by exchange reaction (immuno‐SABER) [24], quantum‐dot SABER [25], barcoded‐antibody based cyclic immunofluorescence (cyCIF) [26], and CO‐Detection by indEXing (CODEX) [27, 28]. These DNA‐based systems have the advantages of a single staining procedure, fast run times, and comparably simple chemistries.

The first version of the CODEX imaging platform was based on iterative rendering of DNA‐conjugated antibodies by DNA polymerase primer extension and fluorescent dNTP analogs [27]. CODEX provides a multiplexed technique that can be easily adopted with minor modifications to regular fluorescent microscopes. However, use of enzymes in the oligonucleotide detection step increased the complexity, background signal, and cost of the method. To simplify and speed up the imaging process, we here updated the technique to use chaotropic solvents that facilitate iterative annealing and stripping of complementary, fluorescently labeled DNA probes to DNA‐conjugated antibodies.

Here, we tested the components of this newly developed CODEX multiparameter imaging method. We validated the specificity and labeling efficiency of 59 unique oligonucleotide barcodes and several dozen antibodies using iterative cycles of annealing, imaging, and stripping using automated fluidics. For this report, the primary antibody panel included major immune cell markers, including CD3, CD4, CD8, CD20, and CD66, functional markers such as CD279 (also known as PD‐1), markers of nonimmune cell populations, and markers of structural features such as vasculature. These antibodies were used to stain five cellularly dense human lymphoid tissues and imaging was performed using the repeated oligonucleotide exchange reaction with the developed CODEX technique. We demonstrate a processing pipeline for aligning cyclic imaging, segmentation of cells in the data, and unsupervised clustering that led to the classification of 31 major cell types. Finally, in a demonstration that the technique can be used to characterize tissue architecture, we used the spatial coordinates of single cells to compare the cell‐type composition of follicles and correlated cell‐cell densities across the human tonsil, spleen, and LN.

In summary, the updated and simplified CODEX platform allows for robust, reproducible identification, and quantification of single cells within complex tissues. This platform enables advanced analysis of intercellular relationships and overarching tissue architecture. Deep, spatially resolved phenotyping of the cellular components in diseases, such as autoimmunity and cancer, will advance our understanding of how spatial features contribute to disease biology and help develop novel diagnostics and therapeutics.

## Results

### CODEX multicycle imaging using DNA oligonucleotide barcodes and automated fluidics

**Figure 1:**
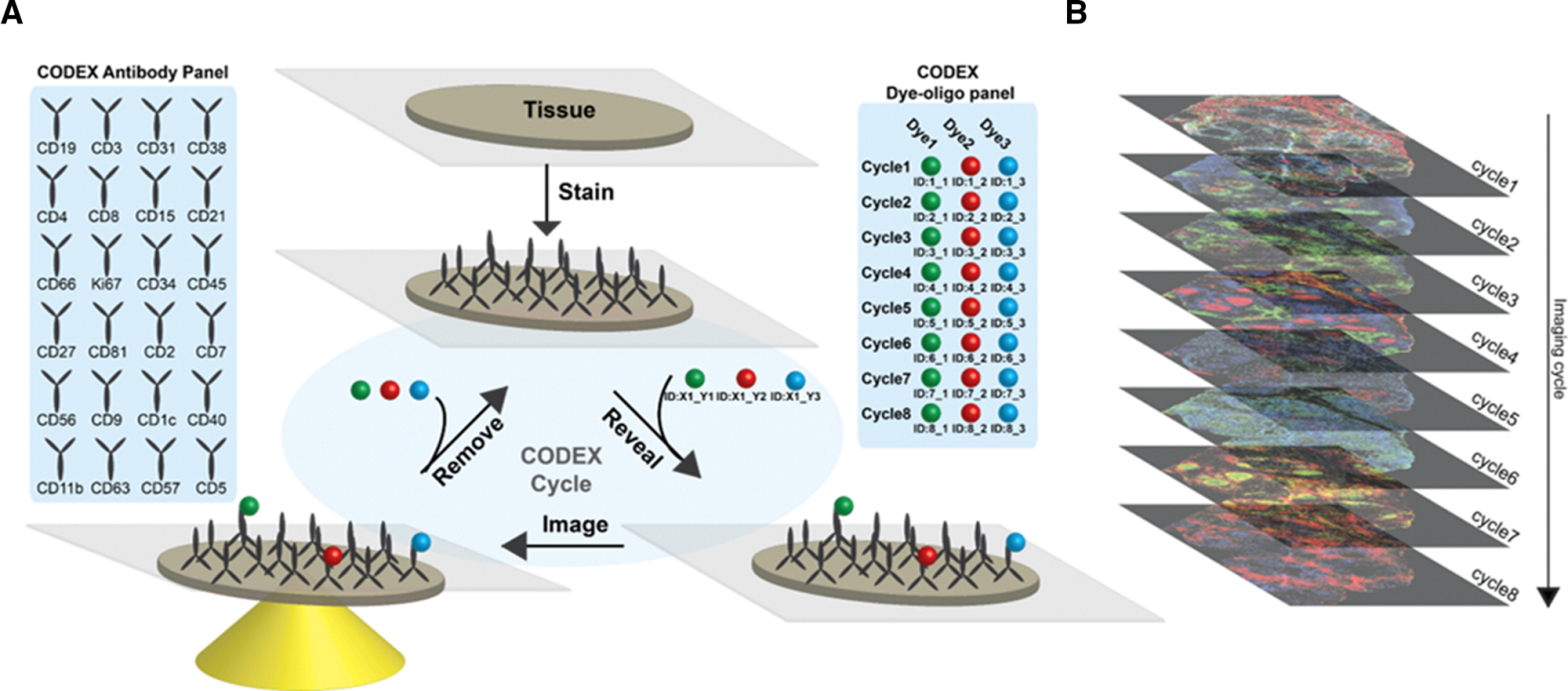
CODEX is a multiplexed tissue imaging technique that relies on antibodies conjugated to unique oligonucleotides. (A) Schematic of repeated oligonucleotide exchange workflow for CODEX imaging. Briefly, antibodies conjugated with unique oligonucleotide barcodes are used to stain a tissue section. Three antibodies bound to the tissue are then rendered visible by adding different complementary fluorescent oligonucleotides. After imaging, the reporter oligonucleotides are stripped through the use of a chaotropic solvent. The cycle is repeated until all antibodies within the panel have been revealed and imaged. (B) Data acquired are then concatenated and processed. The method enables evaluation of up to 60 markers simultaneously.

The approach for the newly adapted CODEX high‐parameter imaging uses iterative cycles of annealing and stripping fluorescently labeled DNA reporter oligonucleotides complementary to DNA barcodes conjugated to antibodies (Fig. 1A). First, antibodies are each labeled with a unique oligonucleotide tag, designed to minimize nonspecific oligonucleotide‐oligonucleotide and oligonucleotide‐tissue binding. Second, the oligonucleotide‐conjugated antibodies are used to stain a tissue section. Third, a robotic fluidics device is used to implement the following fluidics protocol: three fluorescent dye‐conjugated oligonucleotides dissolved in a chaotropic solvent are added to the stained tissue. The chaotropic solvent enables room temperature hybridization of the fluorescent reporter oligonucleotides to their complementary DNA barcodes conjugated to unique antibodies. Fourth, images of the antibody binding events are captured by fluorescent microscopy. Fifth, the three reporter oligonucleotides are removed by changing the solvent, followed by a washing step. The hybridization process is then repeated with three other fluorescent dye‐conjugated oligonucleotides, and this cycle is repeated for as many iterations as necessary to capture the spatial locations of each antibody in the panel (Fig. 1A and B).

The two core components of this technology are (1) the sequence orthogonal barcode library enabling association of each fluorescence signal to a specific antibody clone and (2) the fluidics protocol used to add and remove the fluorescently labeled oligonucleotides in each imaging step. The fluidics protocol is fully automated using off‐the‐shelf components and control software that integrates with the microscope's control software to coordinate the delivery of reagents and image acquisition.

### Validation of CODEX oligonucleotide barcode specificity and annealing/stripping efficiency

**Figure 2:**
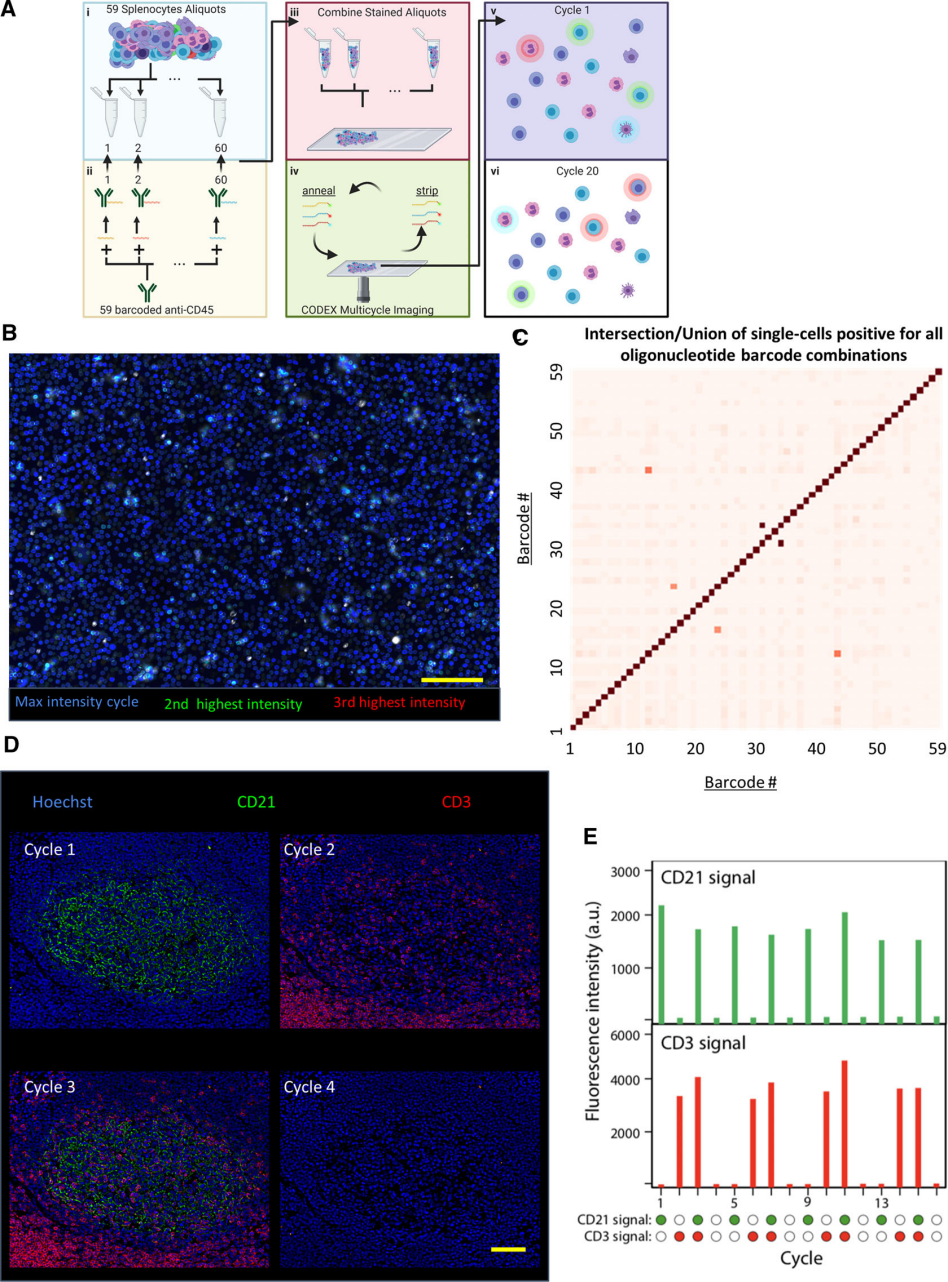
Validation of unique oligonucleotide barcode library. (A) Overall schematic for testing oligonucleotide orthogonality. (i) Fifty‐nine splenocyte aliquots were made, and (ii) each one was stained with anti‐mouse CD45 antibody with a unique oligonucleotide barcode. (iii) All aliquots were pooled and fixed on a slide and rendered visible through CODEX multicycle imaging. In principle, (v) cells should only display one fluorophore and (vi) an individual cell should only be stained in one cycle. (B) Image generated by evaluating each pixel and displaying the highest intensity pixel from all of the cycles with the color blue, the second highest intensity pixel from all of the cycles with the color green, and the third highest intensity pixel from all of the cycles with red (n = 1 experimental replicate, 15 × 10^6^ splenocytes quantified across 59 barcodes). (C) Heatmap of quantified intersection over union of each oligonucleotide pair from single‐cell segmented image data of the anti‐CD45 stained splenocytes for all 59 oligonucleotides. (D) Representative images from CODEX 16‐cycle experiment where oligonucleotide‐conjugated anti‐CD21 (green) was rendered visible in the first cycle, anti‐CD3 (red) in the second cycle, both in the third cycle, and neither in the fourth cycle; the four cycles were repeated three times (n = 1 experimental replicate over 16 channels). (E) Quantification of fluorescent intensities for the two channels across all 16 cycles. Scale bars: 100 μm, 20x magnification.

We created an oligonucleotide library of 59 unique barcodes with sequences selected to eliminate binding between other barcodes or genomic DNA (Supporting Information Table S1). We first evaluated cross‐reactivity of our CODEX barcode oligonucleotide library (Fig. 2A). We aliquoted murine splenocytes into 59 individual tubes and then individually conjugated aliquots of the anti‐mouse CD45 antibody to each barcode and used these barcoded antibodies to stain the 59 different aliquots of mouse splenocytes. After staining, all splenocyte aliquots were combined into a single‐cell spread. The signal for each CODEX barcode channel was revealed by hybridization of up to three complementary fluorescent (FAM, Cy3, or Cy5) oligonucleotides per cycle of CODEX multicycle imaging. If cross‐reactivity between oligonucleotides or tissue is minimal, then there should be low signal from other fluorophores within each cycle and across cycles.

We analyzed the multichannel imaging data visually to evaluate which oligonucleotides exhibit cross‐reactivity with another oligonucleotide. For every pixel in the image, we evaluated the three channels with the greatest measured intensity. We then produced an image where the channel with the highest intensity (for each individual pixel) is represented in blue, the channel with the second highest intensity in green, and the channel with the third highest intensity in red. Because most pixels were blue, this indicated there was minimal oligonucleotide cross‐reactivity (Fig. 2B).

Since each cell population was stained separately, each splenocyte should only be labeled by one barcode. To further quantify possible cross‐reactivity, we segmented single cells as previously reported [27]. Populations of cells were identified that were positive for each barcode sequence by manual gating of the single‐cell expression data. We quantified the overlap (intersection over the union) of oligonucleotide sequences of each pair and plotted this on a heatmap. Only three pairs showed even minimal cross‐reactivity (Fig. 2C). These sequences were not used together in subsequent experiments. These results also demonstrate the efficiency of the annealing and stripping fluidics protocol as fluorescent barcodes from prior cycles were not present in subsequent imaging cycles.

To further validate the CODEX technique, a 16‐cycle imaging experiment was performed using human tonsil tissue stained with barcode‐conjugated antibodies targeting CD21 and CD3. CD21 should only stain B cells and follicular DCs, whereas CD3 should only stain T cells. We first added the dye‐conjugated oligonucleotide complementary to the barcode conjugated to the CD21 antibody and performed imaging. Second, this oligonucleotide was removed, and we added the dye‐conjugated oligonucleotide complementary to that conjugated to the CD3 antibody and performed imaging. Third, this oligonucleotide was removed, and we added dye‐conjugated oligonucleotides complementary to barcodes conjugated to both the CD3 and CD21 antibodies and performed imaging. In the fourth cycle, these were removed, no dye‐conjugated oligonucleotide was added, and the tissue was imaged (Fig. 2D). These fluidics cycles were repeated three more times (for a total of 16 cycles) and the signal associated with each marker was measured. The fluorescence intensity associated with each marker was reduced to background levels after each cycle and the signal generated from the addition of CODEX reporters was reproducible (within 20% of signal intensity) across additional cycles (Fig. 2E).

### CODEX multicycle imaging of dense human lymphoid tissues with a 46‐marker antibody panel

**Figure 3:**
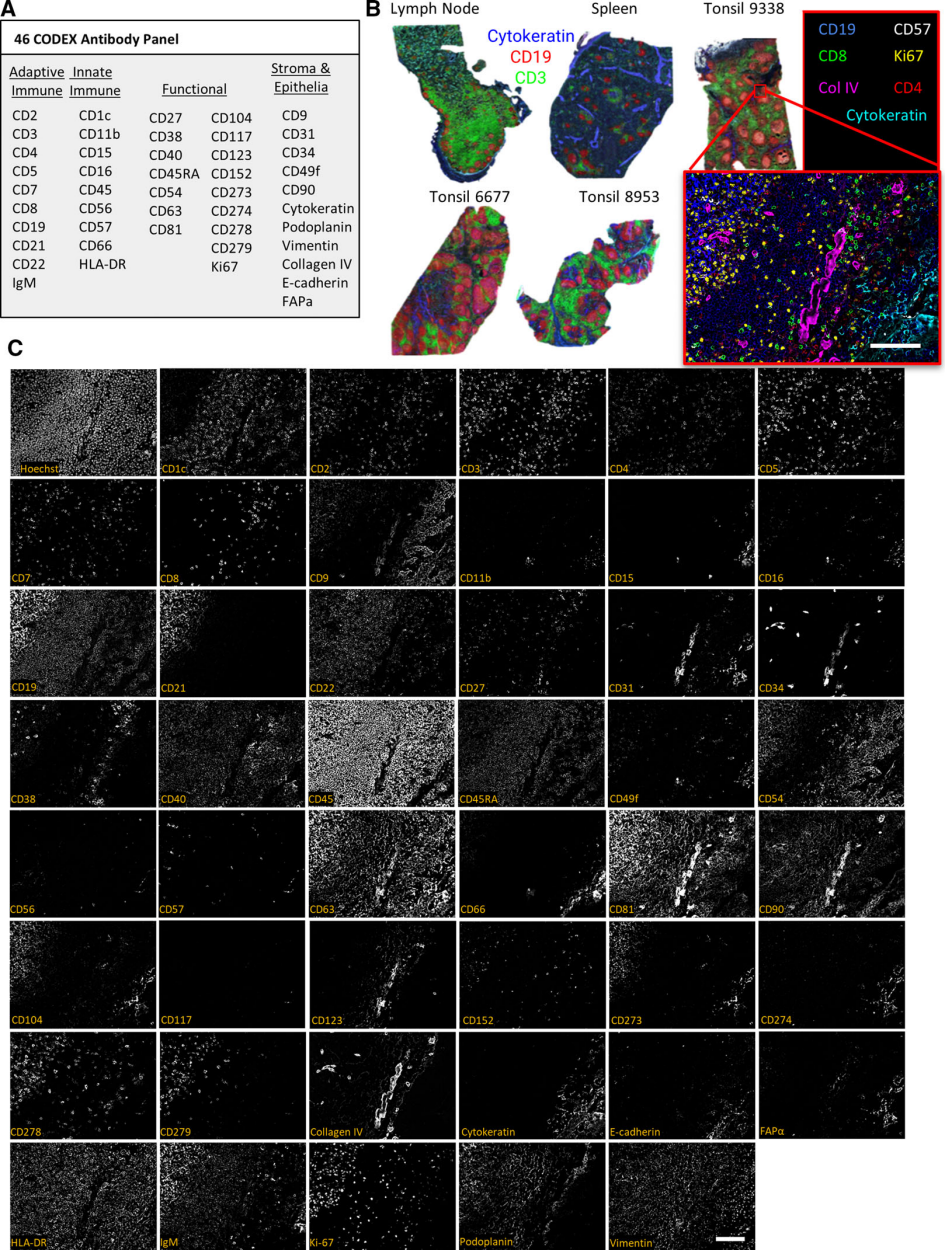
Use of the CODEX protocol to stain cellularly dense human lymphoid tissues with a panel of 46 antibodies (n = 1 CODEX multicycle imaging of each tissue with stated antibody panel: 3 tonsils, each from a different donor, 1 spleen from another donor, and 1 lymph node from another donor). (A) The 46‐antibody panel contains antibodies to key adaptive and innate immune, functional, and stromal, and epithelial markers. (B) Overview images of five lymphoid tissue samples with Cytokeratin (blue), CD19 (red), and CD3 (green) shown. The higher magnification tile image shows tonsil 9338 with CD19 (blue), CD57 (grey), CD8 (green), Ki67 (yellow), Collagen IV (magenta), CD4 (red), and Cytokeratin (cyan). (C) Representative images of a zoomed in tile from tonsil 9338 of all markers and Hoechst staining. Scale bars: 100 μm, 20× magnification.

After validating both the oligonucleotide barcode library and the automated fluidics protocol for CODEX imaging, 46 antibodies were conjugated to unique CODEX barcodes (Supporting Information Table S2). The antibodies chosen target markers of major immune and nonimmune cell populations as well as structural features such as vasculature (Fig. 3A).Each antibody conjugate was tested separately with tissues known to express the marker of interest for validation prior to the multicycle experiment.

This antibody panel was used to stain five fresh‐frozen human tissue sections (3 tonsils, 1 LN, and 1 spleen). We imaged areas of approximately 0.5 cm^2^ per tissue but retained single‐cell resolution by stitching together overlapping imaged tiles (Fig. 3B). Representative images from each of the 46 antibody channels and a nuclear channel (Hoechst 33342) are shown in an example for one tile from tonsil 9338 in Fig. 3C. Expected colocalizations of markers across the B‐cell follicle, epithelium, and T‐cell zone regions of the tissue were observed for each stained channel independent of the imaging cycle in which the marker was detected.

### Single‐cell analysis of CODEX multicycle imaging of human lymphoid tissue

**Figure 4:**
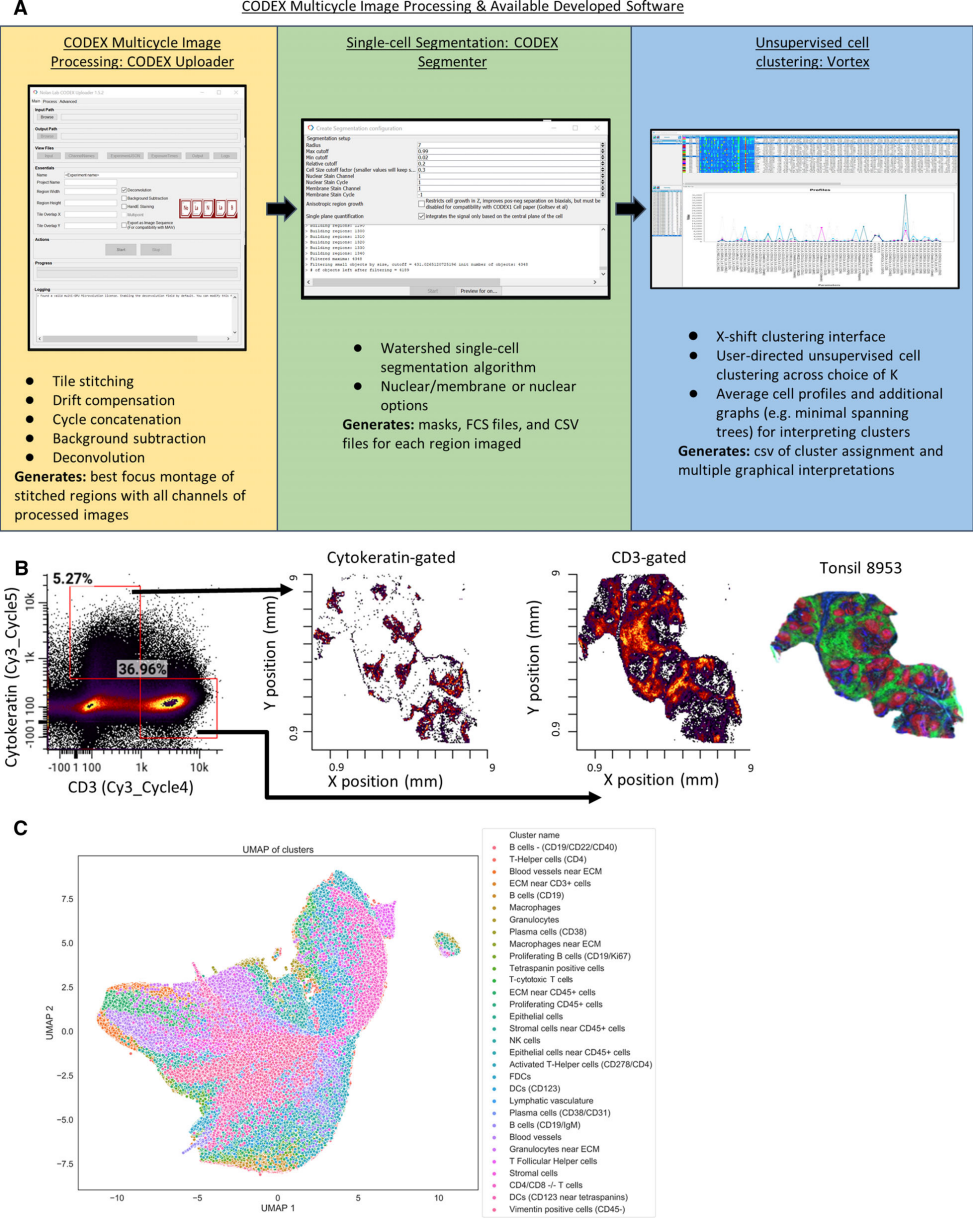
Processing of CODEX multiparameter imaging data. (A) The image analysis was performed using our “CODEX Uploader” for image processing, “CODEX segmenter” for cell segmentation, and “VORTEX” for unsupervised single‐cell clustering. (B) Example of segmented data from tonsil 8953 as an fcs file with populations gated on fluorescent intensity of CD3 or Cytokeratin (no prior gating). These gated CD3^+^ and Cytokeratin^+^ populations are plotted with × (mm), y (mm) coordinates to reveal spatial locations of the populations in the tissue. (C) UMAP plot of all data with the 31 cell types identified by unsupervised clustering indicated by color (total number of cells quantified from single‐cell segmentation from all tissues imaged = 2.3 × 10^6^ cells).

To process the single‐cell multiparametric imaging data, we developed software to align cyclic images, perform deconvolution, subtract background signal, segment single cells, and perform unsupervised single‐cell clustering. This software is available for download at https://github.com/nolanlab/CODEX and https://github.com/nolanlab/vortex and currently supports images generated from Keyence and Zeiss microscopes. The “CODEX Uploader” is used for automated image stitching, deconvolution, drift compensation, background subtraction, and cycle concatenation. The “CODEX Segmenter” enables automated single‐cell segmentation using a watershed algorithm. The “VorteX” interface is used to apply the previously described X‐shift unsupervised clustering [29] (Fig. 4A).

In addition to imaging up to three antibodies per cycle across three fluorescent channels (FAM, Cy3, and Cy5), we also measured the nuclear signal from Hoechst dye during every cycle for image registration. The nuclear stain and the cell membrane marker CD45 were used together to segment individual cells. This generated a multiparameter dataset consisting of fluorescence intensity readings for each of the 46 antibody channels on 2.3 million individual cells in this experiment. The dataset generated is comparable in form to that resulting from mass cytometry or multiparameter flow cytometry, but the spatial coordinates of each segmented cell are retained. Figure 4B shows two of the 46 markers, cytokeratin and CD3, in a traditional flow cytometry‐like plot. Each gated population can then be visualized by X/Y coordinates. In this example, the populations correctly differentiate between epithelial and T‐cell‐rich regions of the tonsil.

With 46 proteins measured simultaneously, manual gating of cell populations is tedious and can be user biased. Consequently, we performed unsupervised clustering analysis using the X‐shift algorithm [29]. In total, 31 clusters were identified, and cell subtypes were assigned based on expression of key markers (Fig. 4C, Supporting Information Figs. S1 and 2). The identified cell types correspond to all major cell subsets (immune, stromal, vasculature). The antibodies in our panel were selected to enable identification of immune cell subsets, and clusters corresponding to T cells, B cells, macrophages, DCs, and NKCs were identified. Furthermore, this integrated pipeline also resolved key functional subpopulations such as plasma cells, T‐follicular helper cells, and activated T‐helper cells. Only a subset of cells displayed relatively few identifying markers, such as the cluster‐labeled tetraspanin positive cells, which were positive for CD81 and CD9, likely a stromal cell subset. In the future, additional stromal cell markers could be included to distinguish these cellular identities. The immune cell subsets were observed at similar frequencies across lymphoid tissues, with higher percentages of innate immune cells like macrophages found in the spleen than in other tissues (Supporting Information Figs. S1 and S2), as expected.

### Characterization of cell‐type distribution in and around B‐cell follicles across lymphoid tissues

The spleen, the LN, and the tonsils are all secondary lymphoid organs that are main sites of immunity. Structurally and functionally, each is distinct, but these organs all have B‐cell follicles, which are critical for antibody responses. Using our multiparameter dataset, we investigated the cell‐type compositions of the B‐cell follicles of the different tissues.

**Figure 5:**
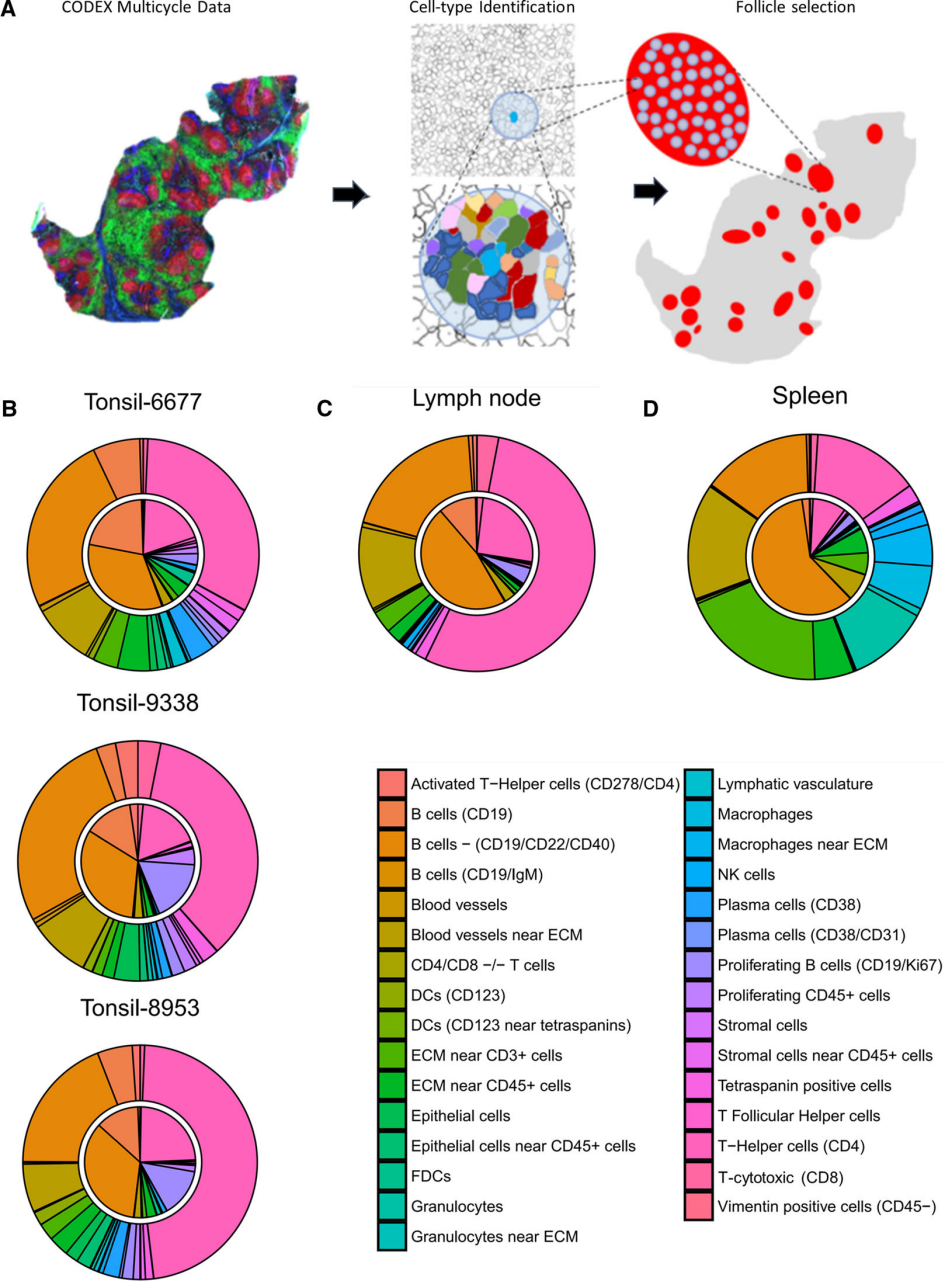
Cell‐type compositions within and surrounding lymphoid tissue follicle structures. (A) CODEX multiparameter imaging data were used to perform unsupervised clustering to identify key subtypes. CD19 expression data were used to manually identify follicles within tissues. Cell‐type compositions of follicle areas and of cells directly surrounding the follicles were analyzed using these masks. (B‐D) Average cell‐type compositions of follicles from (B) three tonsil tissues, (C) a lymph node, and (D) a spleen; the inner pie circle corresponds to the area within each identified follicle and the outer rim corresponds to the cells directly surrounding the follicle (a diameter of 1.2 times the size of a cell).

We identified follicle regions based on CD19 expression and also using tissue morphology based on Hoechst nuclear staining within images of each tissue type (Fig. 5A). The relative distributions of cell types across each follicle were averaged for each tissue, represented by the inner circle of the pie chart (Fig. 5B). The relative distribution of each of these cell types surrounding the follicle regions were calculated, defined as a perimeter of 1.2 times the maximum distance between cells within the segmented follicle region, as represented by the outer circle of the pie chart.

Comparing the follicles of the three tonsils imaged, intrafollicular cellular distributions were similar with 20‐30% CD4^+^ T cells, 60‐70% B cells, and about 10% other cell and stromal cell types (Fig. 5B, inner circle), as expected. However, tonsils 9338 and 8953 had higher proportions of proliferating B cells, than tonsil 6677, and tonsil 6677 had a higher proportion of resting B cells, suggestive of potentially active immune responses within tonsils 9338 and 8953. The cell frequencies in the areas surrounding the follicles of tonsils were similar between tonsil samples. However, there were higher proportions of CD4^+^ T cells (40‐50%), lower proportions of B cells (∼33%), and more blood vessels and blood vessel cells near the ECM (∼20%) than within the follicles (Fig. 5B, outer circle). Additionally, proliferating B cells were present at significantly lower proportions (1‐2%) in the areas surrounding the follicles than within follicles across all tonsils.

Comparison of the follicles from different secondary lymphoid organs revealed considerable divergence in cellular compositions. Follicles from tonsils were more cellularly comparable to follicles from the LN (Fig. 5C) than those from the spleen (Fig. 5D). There was a higher proportion of CD19^+^/CD22^+^/CD40^+^ B cells within follicles of spleen (∼66%) than within follicles of tonsils and LN (33‐50%) (Fig. 5B‐D, inner circle). Additionally, the proportion of helper T (CD4^+^) cells within the splenic follicles (∼10%) was less than those of tonsil and LN (20‐30%) (Fig. 5B‐D, inner circle).

Differences were even larger in proportions of cell populations surrounding follicles of different secondary lymphoid organs. For example, helper T (CD4^+^) cells made up about 33% of cells surrounding follicles in tonsils (Fig. 5B, outer circle), approximately 66% in LN (Fig. 5D, outer circle), and about 20% in the spleen (Fig. 5D, outer circle). Moreover, the areas outside the follicle regions in the spleen contained higher proportions of granulocytes, macrophages, and T cells near ECM than in either the LN or tonsils (∼50% vs. 10‐15%) (Fig. 5B‐D, outer circle).

### Correlated cell‐type densities within follicle regions

Beyond calculating the cellular compositions of distinct regions, spatially resolved cell‐type identification enables assessment of the local densities of various cell types. Under the assumption that cell types move along density gradients, cell types with correlated local densities could indicate that a common molecular program drives positioning, whereas anticorrelated cell‐type densities could correspond to a molecular program that maintains separation.

**Figure 6:**
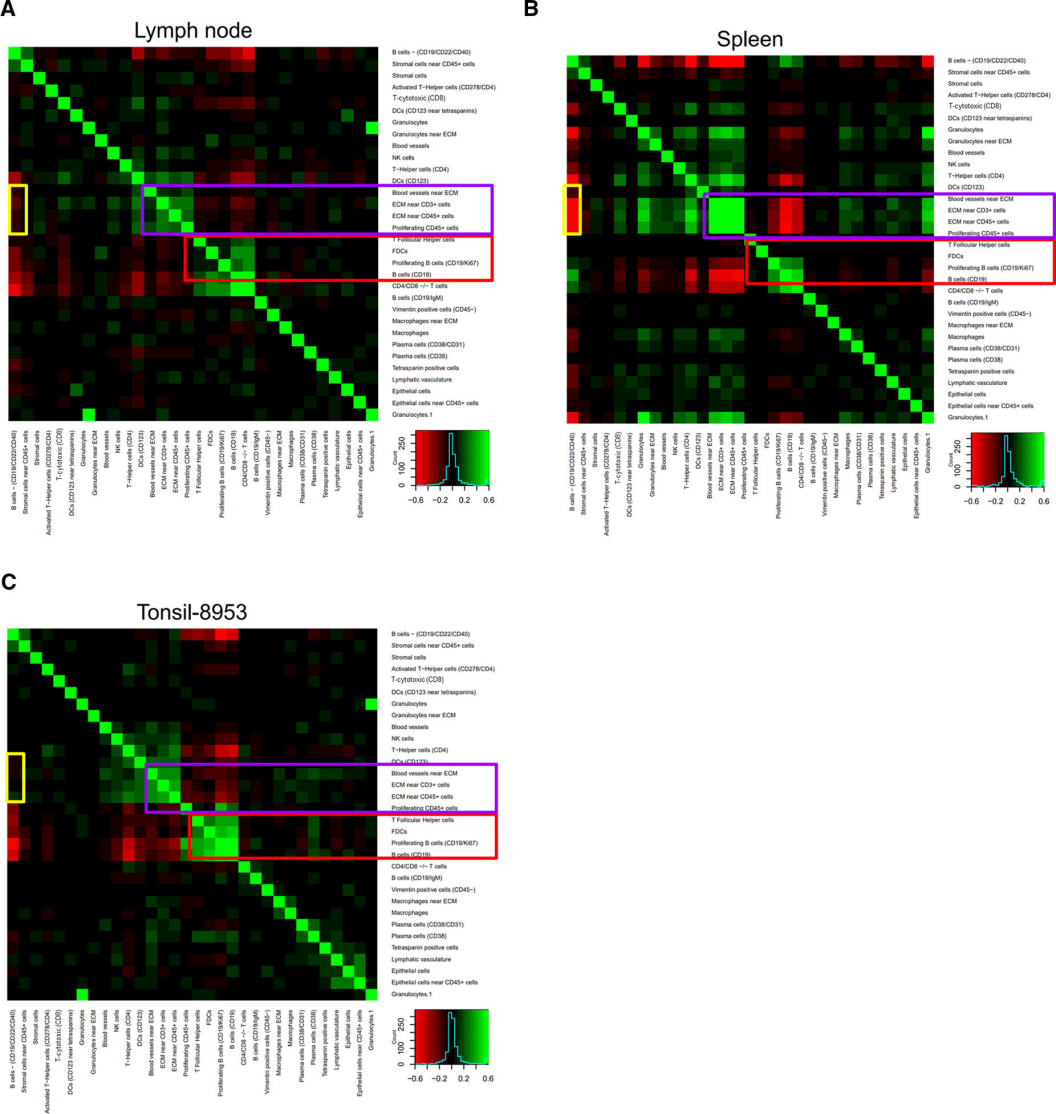
Cell‐cell density correlation analysis for (A) lymph node, (B) spleen, and (C) tonsil determined by evaluating 100 × 100 pixel regions. The heat map scale is from red (anticorrelated) to green (correlated). Correlated modules present in all three tissues are indicated by purple and red boxes; an anticorrelated module present in spleen but not lymph node or tonsil is indicated by the yellow boxes.

We randomly extracted regions of 100 by 100 pixels across each follicle region for each tissue and computed the frequencies of cell types in each of these regions. We then evaluated correlations in these frequencies (Fig. 6, Supporting Information Fig. S3). In all three tissue types, there are two modules of cell types with strongly correlated densities. The first consists of proliferating B‐cell populations, CD19^+^ B cells, follicular DCs, and helper T cells (Fig. 6, red boxes). This module is consistent with the previously described composition of the light zone of the B‐cell GC [30, 31].

The other module of highly correlated cell densities that was common to all three tissue types consisted of collagen IV^+^ blood vessels and CD3^+^ cells and CD45^+^ cells that were near ECM (Fig. 6, purple box). This vascular module was anticorrelated with the light zone module. This also showed that the CD22^+^/CD40^+^ B cells were anticorrelated with this vasculature module in the spleen, whereas this was largely not the case for the LN or tonsil (yellow box). As CD22 and CD40 are markers of marginal zone B cells [32], this anticorrelation likely corresponds to marginal zone structure within the spleen [33].

## Discussion

The streamlined version of multiplexed CODEX imaging reported here uses oligonucleotide‐barcoded antibodies to stain tissues and iterative annealing and stripping of fluorescently labeled reporter oligonucleotides to detect antibody binding. The repeated oligonucleotide exchange reactions eliminate the need for enzymatic reactions to detect antibody binding, decreasing the complexity, time required, cost, computational analysis, and background signal associated with the original method [27]. This is enabled by using DMSO‐based buffers, which act as chaotropic solvents that reduce the melting point of DNA such that annealing and stripping of the DNA fluorescent barcodes can be done at room temperature [34]. Magnesium (Mg^2+^) ions also counteract the negative charges of DNA and improve duplex stability [35, 36]. The method employs a robotic fluidics system that can be integrated with a fluorescent microscope, which will facilitate adoption by laboratories that use traditional immunofluorescent imaging.

The 59 oligonucleotide barcodes designed were not cross‐binding, bound specifically to cells, and were efficiently hybridized and removed using chaotropic solvents. Oligonucleotide barcode design is scalable, and the number of barcodes could be expanded in the future to enable more than 59 markers to be identified simultaneously. For instance, additional fluorophores beyond the three deployed here each cycle could expand the panel size in a given time frame or reduce the time for analysis of a given panel size depending on how the probes are deployed. Validation of additional barcodes would allow antibodies to be used in multiple antibody panels without the need for barcode reconjugation.

Using the repeated oligonucleotide exchange reactions method overcomes two challenges faced in multiplexed fluorescent imaging. First, compared to other multiparameter fluorescent imaging techniques, this method has less spectral overlap as fewer fluorophores are used per cycle. Second, batch effects are minimized by staining with all antibodies at once and using the barcodes to render each antibody fluorescent.

Here, we applied the modified CODEX technique to analyze fresh frozen human immune organ sections. This technique is equally applicable to other clinical specimens, including formalin‐fixed, paraffin‐embedded tissue with minor modifications to the staining protocol, enabling large retrospective analyses through the imaging of tissue microarrays [28]. Moreover, using 3D tissue clearing techniques or expansion microscopy, this method could be applied to image multiple parameters within larger 3D volumes or focused super‐resolution of a single cell [37, 38, 39].

We stained five cellularly dense lymphoid tissues, including samples from tonsil, LN, and spleen, with a panel of 46 barcoded antibodies. Image processing and cell segmentation generated datasets analogous to those generated by multiplexed flow cytometry or mass cytometry. Of course, in addition, the data from this modified CODEX protocol preserve cellular spatial information—a key need to understand tissue architecture and function as cellular communication often involves cell‐cell contact.

Linking spatial positions with multiplexed protein expression data enabled unique spatial single‐cell analysis including unsupervised cell identification, comparison of conserved structures across organs, and analysis of cell density interactions. Comparing across lymphoid organs, highlighted previously established cell types and structures [30, 31, 33]. Analysis of the periphery of B‐cell follicles illustrated that more innate immune cell types were located around splenic follicles than follicles from the LN or tonsil. Some of these differences between tissues observed could be attributed to differences in the number of primary follicles observed, as well as anatomical peculiarities of the spleen. In the future, separating out follicles with or without germinal centers may aid in more directly comparing follicular structure across human lymphoid tissues. Finally, cell‐density analyses revealed conserved structures by cell‐type associations such as the light zone of the B‐cell follicle and the marginal zone of the spleen.

We envision expansion upon the computational spatial analyses described here. For example, one could use morphologic features of segmented cells, analyze neighborhoods of cells, compare and correlate with conventional images using machine learning, and apply algorithms already used within geospatial analyses. Combined with use of this newly developed CODEX protocol will enable novel biological insights into tissue homeostasis, cellular biology, and mechanisms of disease progression.

## Materials and methods

### Materials

**Table:** 

Reagent or Resource	Source	Identifier
Antibodies		
Anti‐Human CD38 (clone HB‐7)	Biolegend	Cat.# 356602
Anti‐Human Vimentin (clone RV202)	BD Biosciences	Cat.# 550513
Anti‐Human CD8 (clone SK1)	Biolegend	Cat.# 344702
Anti‐Human CD15 (clone HI98)	Biolegend	Cat.# 301902
Anti‐Human CD21 (clone Bu32)	Biolegend	Cat.# 354902
Anti‐Human CD66 (clone B1.1/CD66)	BD Biosciences	Cat.# 551354
Anti‐Human HLA‐DR (clone L243)	Biolegend	Cat.# 307651
Anti‐Human CD7 (clone CD7‐6B7)	Biolegend	Cat.# 343102
Anti‐Human CD45RA (clone HI100)	Biolegend	Cat.# 304102
Anti‐Human CD9 (clone HI9a)	Biolegend	Cat.# 312102
Anti‐Human CD19 (clone HIB19)	Biolegend	Cat.# 302202
Anti‐Human CD22 (clone HIB22)	BD Biosciences	Cat.# 555423
Anti‐Human CD90 (clone 5E10)	Biolegend	Cat.# 328102
Anti‐Human CD3 (clone UCHT1)	Biolegend	Cat.# 300402
Anti‐Human Pan‐Cytokeratin (clone AE1/AE3)	Biolegend	Cat.# 914204
Anti‐Human CD5 (clone UCHT2)	Biolegend	Cat.# 300602
Anti‐Human CD273 (clone 24F.10C12)	Biolegend	Cat.# 329602
Anti‐Human Collagen IV (polyclonal)	Abcam	Cat.# ab6586
Anti‐Human CD57 (clone HCD57)	Biolegend	Cat.# 322302
Anti‐Human CD278 (clone C398.4A)	Biolegend	Cat.# 313502
Anti‐Human CD34 (clone 561)	Biolegend	Cat.# 343602
Anti‐Human IgM (clone MHM‐88)	Biolegend	Cat.# 314502
Anti‐Human CD56 (clone HCD56)	Biolegend	Cat.# 318302
Anti‐Human CD45 (clone HI30)	Biolegend	Cat.# 304002
Anti‐Human CD63 (clone H5C6)	Biolegend	Cat.# 353014
Anti‐Human CD1c (clone L161)	Biolegend	Cat.# 331502
Anti‐Human CD31 (clone WM59)	Biolegend	Cat.# 303102
Anti‐Mouse Ki67 (clone B56)	BD Biosciences	Cat.# 556003
Anti‐Human CD123 (clone 7G3)	BD Biosciences	Cat.# 554527
Anti‐Human CD152 (clone BNI3)	Biolegend	Cat.# 369602
Anti‐Human CD2 (clone RPA‐2.10)	BD Biosciences	Cat.# 555324
Anti‐Human CD81 (clone 5A6)	Biolegend	Cat.# 349502
Anti‐Human CD4 (clone A161A1)	Biolegend	Cat.# 357402
Anti‐Human podoplanin (clone NC‐08)	Biolegend	Cat.# 337002
Anti‐Human CD54 (clone HA58)	BD Biosciences	Cat.# 555510
Anti‐Human CD117 (clone YB5.B8)	BD Biosciences	Cat.# 555713
Anti‐Human CD279 (clone EH12.2H7)	Biolegend	Cat.# 329941
Anti‐Human CD274 (clone 29E.2A3)	Biolegend	Cat. # 329702
Anti‐Human Ecadherin (clone 24E10)	Cell Signalling Technology	Custom
Anti‐Human FAPa (clone F11‐24)	eBioscience	Cat. # BMS168
Anti‐Human CD49f (clone GOH3)	BD Biosciences	Cat.# 555734
Anti‐Human CD16 (clone 3G8)	BD Biosciences	Cat.# 555404
Anti‐Human CD11b (clone ICRF44)	BD Biosciences	Cat.# 555386
Anti‐Human CD40 (clone HB14)	Biolegend	Cat.# 313002
Anti‐Human CD27 (clone M‐T271)	BD Biosciences	Cat.# 555439
Anti‐Human CD104 (clone 450–9D)	BD Biosciences	Cat.# 555721
Anti‐Mouse CD45 (clone 30‐F11)	Biolegend	Cat.# 109802
Biological Samples		
Fresh‐frozen human tissues (tonsil, lymph node and spleen)	Deidentified human tissue sections obtained either commercially or from the Stanford Cancer Center Tissue Bank.	N/A
Mouse spleen cells	Spleen cells collected from a healthy mouse	N/A
Chemicals and reagents		
Hoechst 34580	Thermo Fisher Scientific	Cat.# H21486
Coverslips	Electron Microscopy Sciences	Cat.# 72204‐01
Polylysine solution	Sigma Aldrich	Cat.# P8920‐500ML
1X DPBS	Thermo Fisher Scientific	Cat.# 14190250
BS3	Thermo Fisher Scientific	Cat.# 21580
Antibody stabilizer solution	Thermo Fisher Scientific	Cat.# nc0436689
Bio‐Gel P‐30 Gel	Bio Rad	Cat.# 1504150
Software and Algorithms		
R	R Core Team, 2017	https://www.r-project.org
X‐Shift	Samusik, 2016	N/A

   

### Contact for reagent and resource sharing

The image processing and data analysis tools presented in this article are available at https://github.com/nolanlab. Further information will be provided by the corresponding authors upon reasonable request form.

### Methods

#### Tissue slicing

Deidentified fresh frozen tissue samples were obtained from the Stanford Cancer Center Tissue Bank. Tissue sections were placed in OCT after resection and frozen and stored at −80°C. Tissues were sliced using a Leica CM3050 S configured cryostat. OCT embedded tissue samples were mounted to specimen chucks using OCT and equilibrated to the chamber temperature of the cryostat for 20 min. The specimen and surrounding temperatures were approximately −15°C. Tissue sections ranged between 5 and 10 μm. Sliced tissues were placed on polylysine coated coverslips and stored at −80°C until use.

#### Mouse spleen cell extraction

Spleen cells were isolated from C57BL/6 mice. The spleen was placed in a solution of 1X Dulbecco's phosphate‐buffered saline (DPBS) and then macerated through a sterile 70‐μm cell strainer with frequent washes with 1X DPBS. Extracted cells were fixed in 2% paraformaldehyde (PFA) for 10 min at room temperature. Fixed cells were stored in 5% DMSO at −80°C until use. Just prior to use, aliquots of cells were thawed and washed once with 1X DPBS.

#### Antibody‐oligonucleotide conjugation

##### Reagents

Purified antibodies were obtained from the vendors listed in the table above. All antibodies were free from carrier proteins and were in solutions containing PBS with low concentrations of sodium azide. Oligonucleotides with a 5’ protected maleimide modification were supplied by Trilink Biotechnologies.

##### Deprotection of oligonucleotide maleimide groups

Lyophilized oligonucleotide (2‐8 mg) was weighed using a high‐precision scale and placed in a 1.7‐mL Eppendorf tube. Toluene was added to the tube (∼1.5 mL) and incubated at 90°C. The oligonucleotide pellet is not soluble in toluene. After 2 h of incubation, the toluene was removed and replaced with fresh toluene and incubated for another 2 h. This toluene was removed and replaced with an additional aliquot of room temperature toluene. This aliquot of toluene was removed, and the oligonucleotide pellet was washed four times with 100% ethanol (∼1.5 mL per wash). The oligonucleotide was dissolved to a final estimated concentration of 10 mg/mL in 2 mM Tris‐HCl, pH 7, 1 mM EDTA, 150 mM NaCl, and 0.02% w/v NaN_3_. The oligonucleotide concentration was measured using a Nanodrop and standard oligo settings.

##### Antibody partial reduction

Concurrent with oligonucleotide deprotection, purified antibody (typically 50 μg) was partially reduced by adding TCEP to a final concentration of 2 mM (from a stock of 0.5 M at pH 7.0) and EDTA to a final concentration of 2 mM (from a stock of 0.5 M at pH 8.0). The antibody concentration was approximately 0.5 mg/mL. The final volume was adjusted to this concentration using 1X DPBS. This antibody solution was incubated at room temperature (∼23°C) for 30 min.

P30 Biogel slurry was prepared according to the manufacturer's instructions in 2 mM Tris‐HCl, pH 7, 1 mM EDTA, 150 mM NaCl, and 0.02% w/v NaN_3_. A 3‐mL aliquot of the slurry was added to the top of a Biospin chromatography column (Bio‐Rad), and the column was placed in a FACS tube and spun down at 900 *g* for 5 min in a swinging bucket centrifuge. The Bio‐Spin chromatography column was placed in a new FACS tube. The P30 Biogel resin bed was slightly dry at the top after this step.

After the 30‐min incubation with TCEP and EDTA, the antibody solution was pipetted on top of the P30 Bio‐gel resin bed. The column was spun down for 5 min at 900 *g*. The antibody solution collected in the FACS tube was transferred to a PCR tube and the volume adjusted to the original volume of the reduction reaction using excess tris‐based buffer. The total NaCl concentration was increased by 0.6 M by addition of 5 M NaCl stock solution. For a standard antibody reduction volume of 100 μL, 13.6 μL of 5 M NaCl was added.

##### Antibody‐oligonucleotide conjugation

The volume corresponding to 0.1 mg of oligo was added to the antibody solution for each 50‐μg scale antibody conjugation reaction. The solution was incubated at room temperature for 2 h. After this time, the solution was transferred to a preblocked (PBS‐tween) 50‐kDa molecular weight cutoff filter. The remainder of the filter volume was filled with high‐salt PBS (1X PBS with 1 M additional NaCl). After centrifuging at 12 000 *g* for 8 min, the flow through was discarded, and the filter was filled with high‐salt PBS (∼0.45 mL). This step was repeated twice. After the final wash, 0.1 mL of antibody stabilizer solution with 0.5 M NaCl and 5 mM EDTA was added to the top of the column. The filter was inverted into a new collection tube and spun down for 2 min at 3000 *g*. The resulting barcoded antibody solution was stored in a screw‐top Eppendorf tube at 4°C. The barcoded antibodies are suitable for use for at least 6 months.

#### Analysis for barcode cross‐reactivity

##### Barcode and antibody preparation

Oligonucleotide barcodes were designed for sequence orthogonality. Each oligonucleotide included a maleimide moiety to purified antibodies according to the protocol listed above, and a CODEX‐tagged dye, which contains a conjugated fluorophore and a sequence that complements the CODEX‐antibody tag. The CODEX antibody tags were ordered from Trilink Biotechnologies and the CODEX‐tagged dyes were ordered from Integrated DNA Technologies (IDT). CODEX‐tagged dyes were synthesized with one of three fluorophores: Alexa488, Cy3, or Cy5. Each CODEX antibody tag was conjugated to 50 μg of anti‐mouse CD45 (clone 30‐F11).

##### Staining of splenocytes for CD45

Aliquots of mouse spleen cells (0.25‐0.5 million cells) were stained with each of the barcoded anti‐CD45 antibodies. Briefly, thawed and washed cells were resuspended in 1X DPBS, 0.5% w/v BSA, 5 mM EDTA, and 0.02% w/v NaN_3_. Rat IgG and sheared salmon sperm DNA were added to the cells to a final concentration of 0.1 and 0.2 mg/mL, respectively. Cells were gently rotated for 10 min at room temperature. An aliquot of barcoded antibody (∼1 μL antibody/100 μL staining solution) was added to each sample and incubated with gentle rotation for 1 to 2 h. Cells were spun down at 600 *g* for 5 min in a swinging bucket centrifuge. The supernatant was removed, taking care not to disrupt the cell pellet, and replaced with 1X DPBS with 0.5 M NaCl (high‐salt PBS). This step was repeated once more. All cell aliquots were combined into a single tube and centrifuged at 600 *g* for 5 min.

##### Splenocyte attachment to slides

The resultant cell pellet was resuspended at approximately 15 million cells/mL (based on original cell density) and 10‐15 μL of the cell slurry was pipetted onto a polylysine coated coverslip. After 10 min, 90 μL 1.6% PFA in high‐salt PBS was added to the droplet on top of the coverslip taking care not to disrupt the adhered cells. The solution was incubated for 10 min at room temperature. The coverslip was washed three times with high‐salt PBS after which 100 μL 2 mg/mL crosslinking reagent BS3 in high‐salt PBS was added, and the coverslip with adhered cells was incubated for 20 min at room temperature. The coverslip was washed three times with high‐salt PBS and either used directly or stored for up to 2 weeks in 0.5 M NaCl, 0.5% w/v BSA, 1X DPBS, and 0.02% w/v NaN_3_ (staining buffer 3) at 4°C.

##### CODEX multicycle imaging

To screen for barcode orthogonality, the coverslip with the mouse spleen cells stained with barcoded antibodies was adhered to a custom acrylic holder using superglue and nail polish to form a sample well. Cells were stained with Hoechst within the sample well for 5 min prior to running the experiment. Solutions containing sets of up to three dye‐tagged oligonucleotides complementary to barcodes were prepared based on the desired order for imaging of barcoded antibodies. Each solution contained 400 nM of each dye‐tagged oligonucleotide, 2.5 μg/mL Hoechst, 0.3 mg/mL sheared salmon‐sperm DNA, and 88‐96% v/v 10 mM Tris, pH 7.5, 10 mM MgCl_2_, 150 mM NaCl, 0.1% Triton X (v/v), and 0.02% NaN_3_.

#### CODEX‐tagged antibody tissue staining protocol

##### Tissue preparation

Human fresh‐frozen lymphoid tissues were sliced 5‐10 μm thick using a Leica CM3050 cryostat and placed on polylysine coated coverslips. Sliced tissues were stored at −80°C until use. Coverslips with tissues were removed from the freezer and placed on drierite beads for 2 min. Coverslips were incubated at room temperature in acetone for 10 min. Samplers were removed from the acetone and coverslips were dried for 2 min. Tissue culture plates (6‐well) were used to incubate the coverslips with tissue samples for all subsequent buffers. Coverslips were transferred to wells containing 0.5% w/v BSA, 1X DPBS, and 0.02% w/v NaN_3_ (staining buffer 1). This step was repeated once. Tissues were fixed for 10 min in a 1.6% PFA in staining buffer 1, after which they were washed twice in staining buffer 1.

##### Antibody staining

Antibody cocktail solutions were prepared in 61 mM Na_2_HPO_4_, 39 mM NaH_2_PO_4_, 50 mM NaCl, 0.25% w/v BSA, 0.5X DPBS, pH 6.8‐7.0, and 0.01% w/v NaN_3_ (staining buffer 2) using between 0.8‐2 μL per antibody for a 200 μL staining solution. Tissues were stained for 3 h at room temperature in a humidity chamber. Tissue sections were washed twice with staining buffer 2 and fixed for 10 min using 1.6% PFA in staining buffer 3. Tissues were washed three times with 1X DPBS and placed in ice‐cold methanol for 5 min. Tissues were again washed in 1X DPBS and then fixed using 0.2 mL of 2 mg/mL BS3 in high‐salt PBS. Finally, tissues were washed three times in 1X DPBS and stored in staining buffer 3 prior to the CODEX multicycle experiment.

##### CODEX multicycle imaging

Stained tissue sections were mounted to a custom acrylic holder using super glue and nail polish. A custom microscope state insert was manufactured that enabled attachment of the acrylic holder to the microscope and delivery and removal of reagents. A 96‐well plate was prepared for each experiment containing cocktails of up to three dye‐tagged oligonucleotides complementary to barcodes at final concentrations of 400 nM in the presence of 150 mM NaCl, 10 mM Tris (pH 7.5), 0.1% w/v Triton X‐100, 10 mM MgCl_2_^.^6H_2_O, and 0.02% w/v NaN_3_ (1X CODEX buffer), 1:600 Hoechst, and 0.5 mg/mL sheared salmon sperm DNA in a volume of 0.25 mL. Solutions containing 1X CODEX buffer and 20% DMSO and 80% DMSO, respectively, were prepared and loaded onto a custom fluidics instrument based on the autosampler provided by MLE.

Microscope imaging was performed using a Keyence BZX700 inverted microscope with filter cubes for the detection of Hoechst, FAM, Cy3, and Cy5. Exposure times for each antibody were calibrated and ranged from 0.05 to 0.5 s. Each CODEX cycle consisted of addition of solution containing up to three dye‐tagged oligonucleotides to the sample followed by a 5‐min incubation and washing of the sample using the 20% DMSO‐based buffer. The tissue sample was then imaged, the dye‐tagged oligonucleotides were removed by washing with 80% DMSO‐based solution. This process was repeated until all of the antibody staining data was collected. The fluidics process and image acquisition are fully automated using a custom Python‐based script. The total time to perform CODEX imaging depends on the size of the region to be imaged and the resolution or magnification settings of the microscope. Here, large regions (>400 tiles at 20× magnification) were acquired, such that imaging time for each cycle was approximately 3.5 h. The washing, annealing, and stripping procedures require about 30 min per cycle regardless of tissue imaging size. Since there were a total of 19 cycles, the total time to run each tissue was approximately 66 h. As such, the chemistry took overall 9.5 h and the imaging of the large area required 56.5 h.

##### CODEX multicycle image processing

We used the CODEX Uploader (https://github.com/nolanlab/CODEX) for automating image stitching, drift compensation, and cycle concatenation. A watershed‐based, single‐cell segmentation algorithm, formalized as the “CODEX Segmenter” (https://github.com/nolanlab/CODEX), was used for automating segmentation of single cells to generate csv and fcs files. The “VorteX” (https://github.com/nolanlab/vortex) interface was used for X‐shift unsupervised clustering as previously described [29]. Markers CD274, Ecadherin, and FAPa were excluded from the unsupervised analysis and cell‐type assignment. Cell types for each of the 31 clusters were assigned based on averaged overall marker expression profiles.

#### Follicle cell‐type composition analysis

We identified and selected follicle regions across tissues based on CD19 expression and also using tissue morphology based on Hoechst nuclear staining. We manually gated masks over the images and then computed the average distribution of cell types across each tissue. We then used a perimeter around the manual masks of 1.2 times the maximum distance between cells to calculate the relative distributions of each cell type surrounding the follicle regions and averaged these across follicles for a tissue.

#### Cell density correlation analysis

We assessed the local frequencies of cell types within 100 × 100 pixel squares sampled randomly across images. We used cell centroid positions extracted from cell segmentation as the default location of each cell type. This yielded a matrix of celltype concentrations across many sampled regions. We then evaluated correlations between densities of each pair of cell types using the Spearman's rank correlation coefficient.

## Author contributions

Conceptualization: J.K.D., N.S., Y.G., and G.P.N.; Methodology: J.K.D., S.B., G.V., N.S., Y.G., and C.M.S.; Hardware and programming: J.K.D., N.S., and Y.G.; Software: S.S.B., V.G.V., N.S., and Y.G.; Validation: J.K.D., S.B., G.V., N.S., and Y.G.; Formal Analysis: J.K.D., S.S.B., G.L.B., J.W.H., and C.M.S.; Investigation: S.S.B., J.W.H., and C.M.S.; Resources: V.G.V., N.S., and Y.G.; Writing ‐Original Draft: S.S.B. and J.W.H.; Writing‐review and editing: J.W.H., C.M.S., and G.P.N.; Supervision: C.M.S. and G.P.N.; Funding acquisition: G.P.N.

## Conflict of interests

J.K.D. is an employee of Akoya Biosciences, Inc. G.P.N. received research grants from Pfizer, Inc., Vaxart, Inc., Celgene, Inc., and Juno Therapeutics, Inc. during the course of this work. N.S., Y.G., and G.P.N. are inventors on US patent 9909167, granted to Stanford University that covers some aspects of the technology described in this article. J.K.D., N.S., Y.G., and G.P.N. have equity in and/or are scientific advisory board members of Akoya Biosciences, Inc. C.M.S. is a scientific advisor to Enable Medicine, LLC. The other authors declare no commercial or financial conflict of interests.

### Peer review

The peer review history for this article is available at https://publons.com/publon/10.1002/eji.202048891

## Supporting information

Supporting information

## Data Availability

The image processing and data analysis tools presented in this article are available at https://github.com/nolanlab. We have uploaded the single‐cell segmented data (https://doi.org/10.6084/m9.figshare.13079291) used in this manuscript on figshare (https://figshare.com/). Further information will be provided upon request from the corresponding authors.
