# Supplementary material for: Highly multiplexed tissue imaging using repeated oligonucleotide exchange reaction
Source: Eur J Immunol. 2021 Mar 10;51(5):1262–77. doi: 10.1002/eji.202048891 (PMC8251877; doi:10.1002/eji.202048891)
Supplement: Supplementary file 1 — Supporting information [file EJI-51-1262-s001.pdf]

<u>Supporting Information</u>

Herein contains Supporting Information Figures 1-3 and Supporting Tables 1-2:

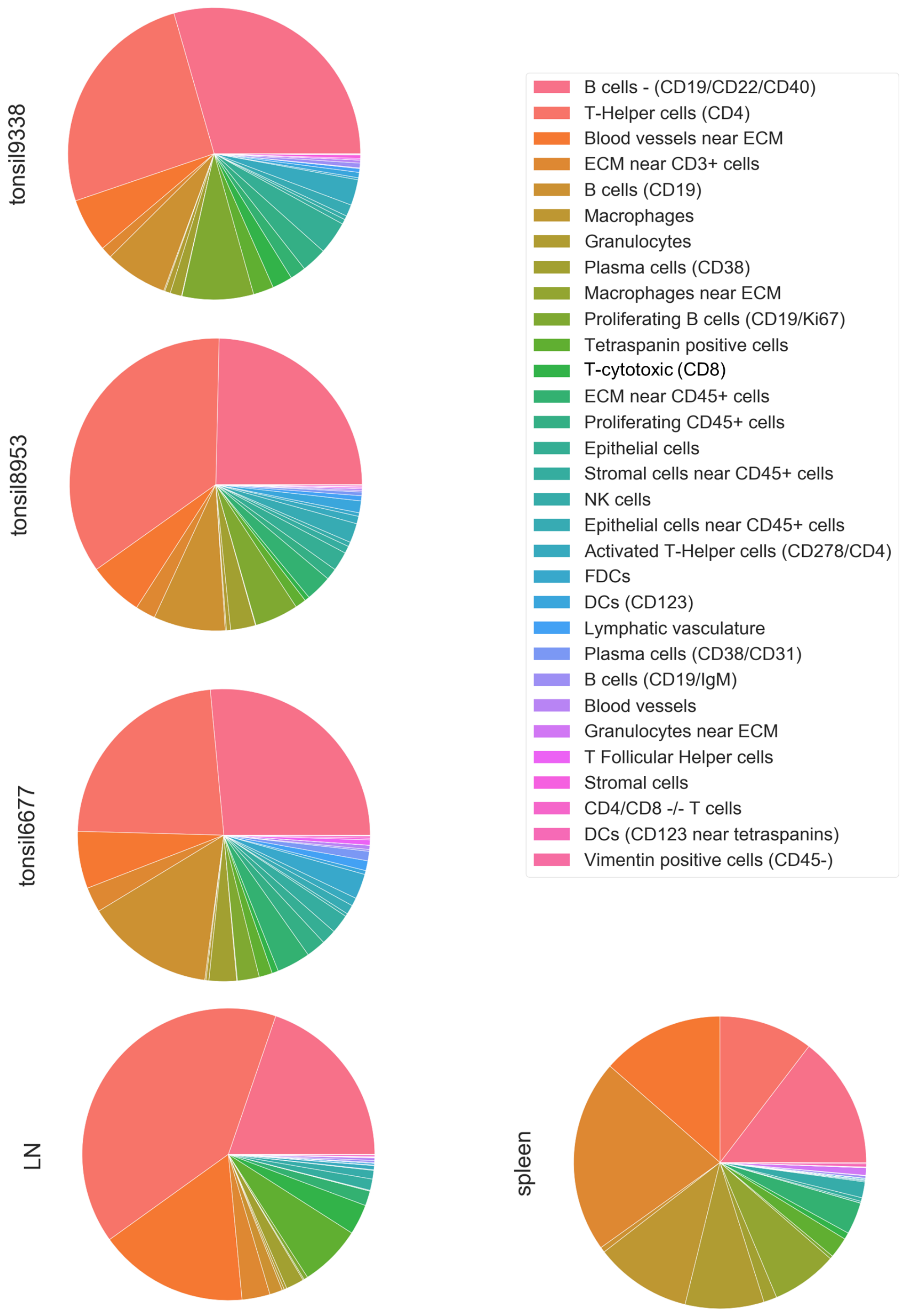

Supporting Information Figure 1: Pie charts of cell-type compositions for all tissues imaged.

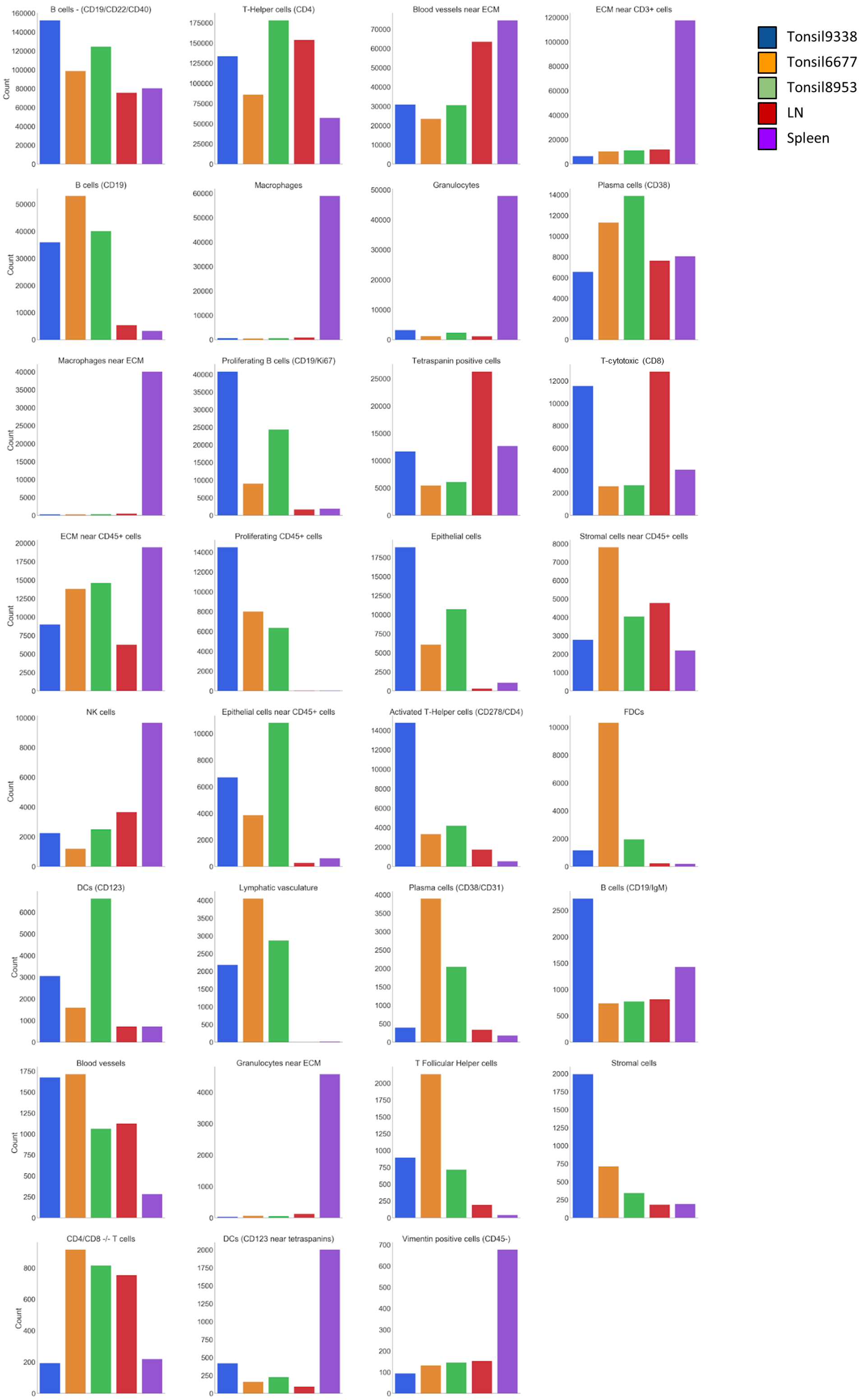

Supporting Information Figure 2: Bar graphs of the numbers of each cell type in all tissues imaged.

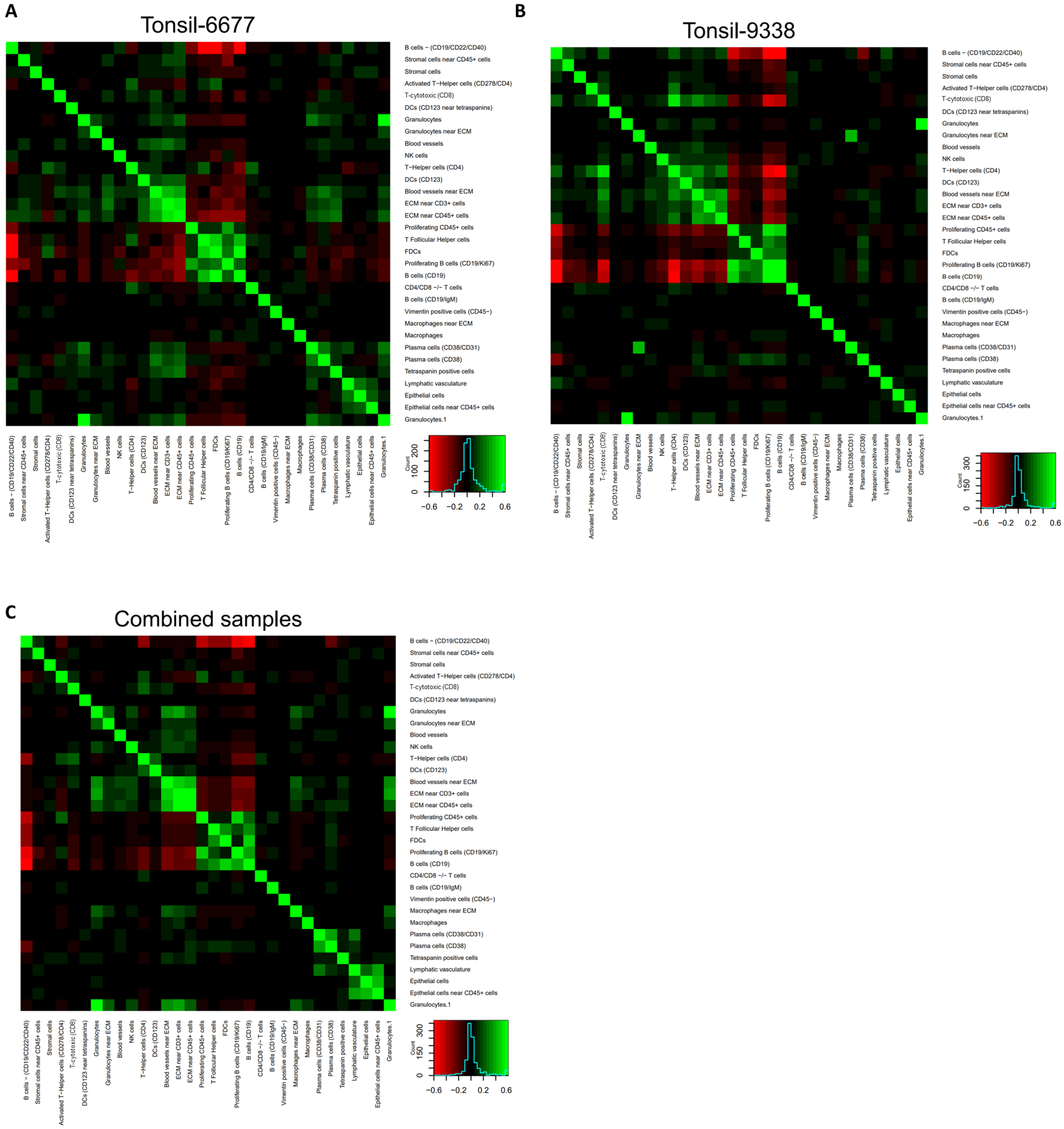

Supporting Information Figure 3: Cell-cell density correlation analysis across lymphoid tissues determined by evaluating 100 x 100 pixel squares for (A) tonsil 6677, (B) tonsil 9338, and (C) all samples combined.

Supporting Table 1: CODEX multicycle panel, order, oligo number, and antibody clones

	CH1	A488			Cy3			Cy5		
Cycle		antibody	oligo	clone	antibody	oligo	clone	antibody	oligo	clone
1	Hoechst	CD38	A2	HIB7	CD19	A0	HIB19	CD31	A1	WM59
2	Hoechst	Vimentin	A4	RV202	CD22	A6	HIB22	Ki67	A3	B56
3	Hoechst	CD8	A5	SK1	CD90	A7	5E10.00E+10	CD123	A9	7G3
4	Hoechst	CD15	A8	HI98	CD3	B0	UCHT1	CD152	B2	BN13
5	Hoechst	CD21	B1	Bu32	cytokeratin	B3	AE1/AE3	CD2	B4	RPA-2.10
6	Hoechst	CD66	C4	B1.1/CD66	collagen IV	B6	poly	CD81	B5	5A6
7	Hoechst	HLA-DR	C7	L243	CD57	B9	HCD57	CD4	B7	A161A1
8	Hoechst	CD7	D8	CD7-6B7	CD278	C2	C398.4A	podoplanin	C0	NC-08
9	Hoechst	CD45RA	E2	HI100	CD34	C3	561	CD54	C1	HA58
10	Hoechst	CD9	E4	HI9a	IGM	D0	MHM-88	CD117	C5	YB5.B6
11	Hoechst				CD56	D1	HCD56	CD279	C8	EH12.2H7
12	Hoechst				CD45	D6	HI30	CD49f	D2	GoH3
13	Hoechst				CD5	D7	UCHT2	CD16	D3	3G8
14	Hoechst				Ecadherin	E6	2.40E+11	FAPalpha	D4	BMS168
15	Hoechst				CD63	E7	H5C6	CD11b	D9	ICRF44
16	Hoechst				CD1c	F2	L161	CD40	E0	HB14
17	Hoechst				CD274	F3	29E.2A3	CD27	E1	M-T271
18	Hoechst							CD104	F5	450-9D
19	Hoechst							CD273	F6	24F.10C12

Supporting Table 2: CODEX Oligonucleotide sequences

Name	Top oligo sequence (5'-3')	dye sequence	Dye	# in cross-validation
**A0**	ATAGCAGTCCAGTTGCTGATGGTTTAGGACTACGG	GTAGTCCTAAACCAT	Cy3	2
**A1**	ATAGCAGTCCAGGATAATAGTACTCCTCGCCGCAA	CGGCGAGGAGTA	A488	3
**A2**	ATAGCAGTCCAGGCCCACTCTCCCATTAGTCGGAA	CCGACTAATGGGAGA	A488	1
**A3**	ATAGCAGTCCAGCACAGGTGGATGTGTTACGATGG	ATCGTAACACATCCA	Cy5	6
**A4**	ATAGCAGTCCAGAGCCCGCTAAGATATTCTAAGTT	CTTAGAATATCTTAGCG	A488	4
**A5**	ATAGCAGTCCAGGCGTAACGCAGATGAATATTCAA	GAATATTCATCTGCG	A488	7
**A6**	ATAGCAGTCCAGATCGAAGGGTTATACACTCGTGG	ACGAGTGTATAACCC	Cy3	5
**A7**	ATAGCAGTCCAGCGATGGAGATTCAGTCTCTCGGG	CGAGAGACTGAATCT	Cy3	8
**A8**	ATAGCAGTCCAGCGAGCACTGTAATAGGCACTATT	TAGTGCCTATTACAG	A488	10
**A9**	ATAGCAGTCCAGGGAACAAAGCGGTCTTACGGTTT	ACCGTAAGACCGCTT	Cy5	9
**B0**	ATAGCAGTCCAGAATCGGATGTAGGATGGTCTCGG	GAGACCATCCTACAT	Cy3	11
**B1**	ATAGCAGTCCAGGTACCGGACGTGCCGTTTCACTT	GTGAAACGGCACG	A488	13
**B2**	ATAGCAGTCCAGCTTCGCGGTTTCCTCAGACACGG	GTGTCTGAGGAAACC	Cy5	12
**B3**	ATAGCAGTCCAGTTAAGAATAAGGGCTCATTGTTT	ACAATGAGCCCTTAT	Cy3	14
**B4**	ATAGCAGTCCAGAGGCAGAGGCACGCCCTTTTAAA	TAAAAGGGCGTGC	Cy5	15
**B5**	ATAGCAGTCCAGCGGATCCACTTGTCTAACCAATT	TTGGTTAGACAAGTG	Cy5	18
**B7**	ATAGCAGTCCAGTATGATTGGTCCACTAACGTAGG	TACGTTAGTGGACCA	A488	24
**B8**	ATAGCAGTCCAGCGCGTGATAGGGCATTTGAAGGG	CTTCAAATGCCCTAT	Cy5	21
**B9**	ATAGCAGTCCAGCCCGTACACATGAGCGAATCAGG	TGATTCGCTCATGTG	Cy3	20
**C0**	ATAGCAGTCCAGAACGACTACCAAATCCTGATG	CATCAGGATTTGGTA	Cy5	27
**C1**	ATAGCAGTCCAGCTATTATCATGAGGAGCGGCG	CGCTCCTCATGATAA	Cy5	30
**C2**	ATAGCAGTCCAGGTGTCCACCTACACAATGCTA	TAGCATTGTGTAGGT	Cy3	23
**C3**	ATAGCAGTCCAGCGCATTGCGTCTACTTATAAG	CTTATAAGTAGACGC	Cy3	26
**C4**	ATAGCAGTCCAGTTACTGTATGAGTAGTAATCT	AGATTACTACTCATACA	A488	16

**C5**	ATAGCAGTCCAGGCTACCTCTAAGTCAGAGAGC	GCTCTCTGACTTAGA	Cy5	33
**C6**	ATAGCAGTCCAGGCTCGTGACATTATCCGTGAT	ATCACGGATAATGTC	A488	19
**C7**	ATAGCAGTCCAGTTTCTCACTACTATTAGTACT	AGTACTAATAGTAGTGA	A488	22
**C8**	ATAGCAGTCCAGCCTTGTTTCGCCAGAATGCCA	TGGCATTCTGGC	Cy5	36
**C9**	ATAGCAGTCCAGCTCTTCCGACTCGAACCTGAG	CTCAGGTTCGAGTC	A488	25
**D0**	ATAGCAGTCCAGCATGGCCTGCACGGCAAAGTG	CACTTTGCCGTGC	Cy3	29
**D1**	ATAGCAGTCCAGATGGCAATAACGCCTCGTATC	GATACGAGGCGTTAT	Cy3	32
**D2**	ATAGCAGTCCAGTCCGTCTCCGTGCGGGAAAAT	ATTTTCCCGCACG	Cy5	39
**D3**	ATAGCAGTCCAGATACTAACCAGGTGATTGCAT	ATGCAATCACCTGGT	Cy5	42
**D4**	ATAGCAGTCCAGGCGCACTATCCCGTAAACTTC	GAAGTTTACGGGATA	Cy5	45
**D5**	ATAGCAGTCCAGTCAAGGCAGGTCAACTCGCAC	GTGCGAGTTGACCT	Cy3	35
**D6**	ATAGCAGTCCAGAGTGAGGGTCACATGGTCGTT	AACGACCATGTGACC	Cy3	38
**D7**	ATAGCAGTCCAGAGAAGTGCGGATTTCGTATTT	AAATACGAAATCCGC	Cy3	41
**D8**	ATAGCAGTCCAGGCAGCTCGTCAGTACTTTCAG	CTGAAAGTACTGACG	A488	28
**D9**	ATAGCAGTCCAGGTGACGCTTATTATGGACTTC	GAAGTCCATAATAAGC	Cy5	48
**E0**	ATAGCAGTCCAGGTGTAAACAAAACTGCTGTCG	CGACAGCAGTTTTGT	Cy5	51
**E1**	ATAGCAGTCCAGGTGAATTCTTATTCCCGAATA	TATTCGGGAATAAGA	Cy5	53
**E2**	ATAGCAGTCCAGGAAGCCTAGGGGAACAGGTTG	CAACCTGTTCCCCTA	A488	31
**E3**	ATAGCAGTCCAGGTTTAATTAGCTTAAGAGAGT	ACTCTCTTAAGCTAATT	A488	34
**E4**	ATAGCAGTCCAGACGTTGATAAATATTTTACAGAGT	ACTCTGTAAAATATTTATC	A488	37
**E5**	ATAGCAGTCCAGACTCGCGATGTACGTGCTTTGGTT	AACCAAAGCACGTA	A488	40
**E6**	ATAGCAGTCCAGAGCGGAGTTGCCGACGACGAAGGC	GCCTTCGTCGTC	Cy3	44
**E7**	ATAGCAGTCCAGCAAGGAGATGCTTCTTGTGGAACC	GGTTCCACAAGAAG	Cy3	47
**E8**	ATAGCAGTCCAGCAATACATACGTGGCCCGGCAGTT	AACTGCCGGG	A488	43
**E9**	ATAGCAGTCCAGCCCCTTGTCCAACCAAACTGACCG	CGGTCAGTTTGGTT	A488	46
**F0**	ATAGCAGTCCAGCCGGTTGAGCGTGTCACCCCCAGC	GCTGGGGGTGA	Cy3	50
**F1**	ATAGCAGTCCAGCGACGGCGCGACAACGCGACGGAT	ATCCGTCGCGTT	A488	49
**F2**	ATAGCAGTCCAGCTCTTATCACATTTGCTTCGACGA	TCGTCGAAGCAAAT	Cy3	52
**F3**	ATAGCAGTCCAGCTGTCGGGGTCGCTTGGGTGTTTA	TAAACACCCAAGCG	Cy3	54

**F4**	ATAGCAGTCCAGGACGCTGGGATCTGTCGGTCGTCA	TGACGACCGACAG	Cy5	55
**F5**	ATAGCAGTCCAGGAGGTCATTTCAACAAATATTGTT	AACAATATTTGTTGAAAT	Cy5	56
**F6**	ATAGCAGTCCAGTCTTAATGAATGGCCGGAAGTGGT	ACCACTTCCGGC	Cy5	57
**F7**	ATAGCAGTCCAGTGGTCCCCAGACGCCGGGGCACCA	TGGTGCCCCG	Cy5	58
**F8**	ATAGCAGTCCAGTTTTCTATCGCAAGGAACTACCGA	TCGGTAGTTCCTTG	Cy5	59
